# Maternal mortality due to cardiovascular disease in the Netherlands: a 21-year experience

**DOI:** 10.1007/s12471-019-01340-w

**Published:** 2019-11-27

**Authors:** H. Lameijer, J. M. Schutte, N. W. E. Schuitemaker, J. J. M. van Roosmalen, P. G. Pieper, Dutch Maternal Mortality and Morbidity Committee

**Affiliations:** 1 Department of Cardiology, University Medical Centre Groningen, University of Groningen, Groningen, The Netherlands; 2 Department of Emergency Medicine, University Medical Centre Groningen, University of Groningen, Groningen, The Netherlands; 3 Department of Obstetrics and Gynaecology, Isala Zwolle, Zwolle, The Netherlands; 4 Athena Institute, VU University, Amsterdam, The Netherlands; 5 Department of Obstetrics, Leiden University Medical Centre, Leiden, The Netherlands; 6 Department of Obstetrics and Gynaecology, Diakonessen Hospital, Utrecht, The Netherlands

**Keywords:** Maternal mortality, Cardiovascular disease, Pregnancy, Death, Heart disease

## Abstract

**Objective:**

Cardiovascular disorders are the leading cause of indirect maternal mortality in Europe. The aim of this study is to present an extensive overview concerning the specific cardiovascular causes of maternal death and to identify avoidable contributing care factors related to these deaths.

**Methods:**

We assessed all cases of maternal death due to cardiovascular disorders collected by a systematic national confidential enquiry of maternal deaths published by the Dutch Maternal Mortality and Morbidity Committee on behalf of the Netherlands Society of Obstetrics and Gynaecology over a 21-year period (1993–2013) in the Netherlands.

**Results:**

There were 96 maternal cardiovascular deaths (maternal mortality rate due to cardiovascular diseases 2.4/100,000 liveborn children). Causes were aortic dissection (*n* = 20, 21%), ischaemic heart disease (*n* = 17, 18%), cardiomyopathies (including peripartum cardiomyopathy and myocarditis, *n* = 20, 21%) and (unexplained) sudden death (*n* = 27, 28%). Fifty-five percent of the deaths occurred postpartum (*n* = 55, 55%). Care factors that may have contributed to the adverse outcome were identified in 27 cases (28%). These factors were patient-related in 40% (pregnancy against medical advice, underestimation of symptoms) and healthcare-provider-related in 60% (symptoms not recognised, delay in diagnosis, delay in referral).

**Conclusion:**

The maternal cardiovascular mortality ratio is low in the Netherlands and the main causes of maternal cardiovascular mortality are in line with other European reports. In a minority of cases, care factors that were possibly preventable were identified. Women with cardiovascular disease should be properly counselled about the risks of pregnancy and the symptoms of complications. Education of care providers regarding the incidence, presentation and diagnosis of cardiovascular disease during pregnancy is recommended.

## What’s new?

Cardiovascular diseases are the leading cause of indirect maternal mortality. We found maternal cardiovascular mortality to be low in the Netherlands, but still significant. We describe the causes of maternal mortality and define care factors that can possibly be avoided in order to improve care. Our data result in several recommendations that may contribute to further improvement of care for pregnant women with cardiovascular diseases.

## Introduction

Cardiovascular disorders (CVD) are the leading cause of indirect maternal mortality (mortality not due to direct obstetric causes) in European countries, including the Netherlands [1–4]. Moreover, in the Netherlands a rise in the indirect maternal death ratio (23% indirect deaths, including cardiac deaths) was observed between 1993 and 2005 [2]. Moreover, an increase in direct maternal mortality was observed, which includes (unexplained) sudden deaths. An ongoing national systematic confidential enquiry into maternal deaths is being performed in the Netherlands by the Dutch Maternal Mortality and Morbidity Committee (MMMC) on behalf of the Netherlands Society of Obstetrics and Gynaecology. Data and conclusions are periodically published (inter)nationally. The enquiry assesses the prevalence and causes of maternal deaths and identifies contributing care factors that may be related to the adverse outcome, with the aim of further reducing mortality and morbidity in the Netherlands. On behalf of the MMMC, the aim of our study is to present an extensive overview concerning the specific cardiovascular causes of maternal death and to identify factors related to the rise in maternal cardiovascular mortality in the Netherlands over a 21-year period [2, 3]. Furthermore, we will address possibilities for improving care in these women and provide recommendations in an attempt to contribute to a reduction of maternal cardiovascular mortality.

## Methods

We used data collected by the MMMC. The members of the MMMC (11 obstetricians and one obstetrically orientated anaesthetist working in the field of maternal foetal medicine, from both university and non-university hospitals) are appointed by the Dutch Society of Obstetrics and Gynaecology. Maternal mortality cases were voluntarily reported to the MMMC by obstetricians and in some cases by midwives and general practitioners. A request to report every death to the MMMC during or within 1 year after pregnancy in the study period was submitted to all 98 obstetric departments in the Netherlands and to the reporter of maternal death of Statistics Netherlands. All maternal deaths in the Netherlands reported to the MMMC during or within 1 year after pregnancy between January 1993 and December 2013 were included in the study. Additional cases were collected yearly after a cross-check with the database of Statistics Netherlands, which collects all vital registration data from the Netherlands. Ethics committee approval was not applicable because the data were derived from an ongoing confidential enquiry.

Maternal death was defined and classified according to the World Health Organisation’s International Classification of Diseases, 10th revision (ICD-10) [5]. Deaths were classified as direct, indirect or fortuitous. A single underlying cause or mode of death was assigned to each case by the members of the MMMC. The underlying cause of death is the disease or injury which results directly in death or initiates the chain of events leading directly to death [6]. The mode of death is the disease or injury that ends life directly. Substandard care was defined as all care factors which may have resulted in suboptimal care and which had a probable negative influence on the chain of events leading to death. This could be assigned to any person involved in the care of pregnant women and to the pregnant woman herself. Avoidance of such factors did not necessarily mean that death would have been prevented. The standard of care was the care as stated in national guidelines [7–12]. If there was no (appropriate) guideline, the best available evidence was used. The anonymised cases were individually assessed for substandard care factors by the members of the MMMC and discussed at a group meeting for a final decision. When a consensus could not be reached, the decision was based on the assessment of the majority of the committee.

A confidential enquiry was completed into each case reported to the MMMC. For each maternal death, data were collected by the MMMC on a standard questionnaire, including information concerning general and obstetric histories as well as the index pregnancy. Sources of information included antenatal charts, laboratory and bacteriological results, pathology and autopsy reports and professional correspondence, if provided. For cases provided by Statistics Netherlands only the cause of death and maternal age could be retrieved.

For the current report, we included all deaths caused by cardiovascular disease (including congenital, valvular and ischaemic heart disease, pulmonary hypertension, cardiomyopathy, arrhythmias, aortic dissection, myocarditis and infective endocarditis). Furthermore, we included all cases of sudden death (sudden unexplained death syndrome (SUDS) and sudden arrhythmic adult death syndrome (SADS), defined as SUDS with negative pathological and toxicological assessment or sudden death in women with a known arrhythmic disease) [13]. We excluded all cases in which maternal mortality occurred more than 6 months after pregnancy (postpartum period) and all cases of mortality due to vascular disease other than aortic or coronary artery disease, such as cerebral vascular accidents or ischaemia of the abdominal arteries.

Obstetric and neonatal complications were defined as diagnosed and treated by the responsible physicians and according to definitions in previous papers and included caesarean section (CS, both planned and emergency), pregnancy-induced hypertension, (pre-)eclampsia, haemolysis elevated liver enzymes and low platelets (HELLP) syndrome, postpartum haemorrhage, preterm labour, early foetal death (intrauterine death ≤20 weeks of gestation, not induced abortion), offspring death (defined as the total number of stillbirths >20 weeks of gestation and deaths up to 6 months postpartum), neonatal respiratory distress syndrome, pre-term birth (<37 weeks of gestation), low birth weight (<2500 g), occurrence of congenital heart disease (CHD) or other congenital disease in the offspring and Apgar score <7 (at 1 and 5 min after birth) [14]. Maternal mortality ratio is calculated per 100,000 live births.

Individual cases, updates and trends have been presented at obstetric meetings in the Netherlands, published in (inter)national journals, national guidelines and as case reports in a Dutch medical journal. Some cases have been included in previous publications concerning vascular dissection and rupture and (indirect) maternal mortality [2, 15].

Statistical analysis was performed using IBM SPSS Statistics Premium′ V 22 for Windows (IBM Corp. Released 2011. IBM SPSS Statistics for Windows, Version 22.0. Armonk, NY: IBM Corp.). Continuous data are presented as means with standard deviation or median with interquartile range or range depending on their distribution. Normality was tested with the Kolmogorov-Smirnov test with Lilliefors’ correction. Absolute numbers and percentages were presented for categorical data. Missing data were excluded from the analysis.

## Results

**Fig. 1:**
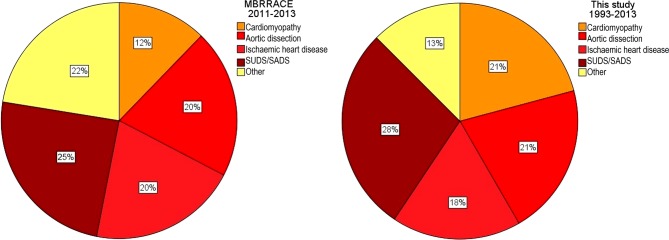
Causes of death in the women who died during pregnancy or in the postpartum period due to cardiovascular diseases in the UK during 2011–2013 according to the MBRRACE-UK study [16] and in this study during 1993–2013 in the Netherlands. In our study cardiomyopathy includes all cardiomyopathies including peripartum cardiomyopathy and myocarditis

From January 1993 until December 2013, 96 women in the Netherlands died from cardiovascular diseases during their pregnancy. This results in a maternal mortality ratio (MMR) from cardiovascular diseases of 2.4 per 100,000 liveborn children. Baseline characteristics, timing and causes of death of the women who died are provided in Fig. 1 and Tab. 1. Fifty-five percent of the women died during the postpartum period. Care factors contributing to the adverse outcome were reported in 27 cases (28%) (Tab. 2). These factors were completely or partially attributable to the patient in 40%. There was no direct overall relation to ethnicity (Dutch vs non-Dutch, *p* = 0.63). However, when evaluating maternal mortality per liveborn neonate, maternal death occurred more frequently in women with a Dutch-Antillean (MMR 11.25) or Surinamese (MMR 5.92) ethnicity compared to Dutch (MMR 2.17), Moroccan (MMR 0.75) or Turkish (MMR 2.59) ethnicity. Non-Dutch ethnicity itself did not differ from Dutch ethnicity (MMR 2.11 vs 2.17). Avoidable care factors contributing to the death had a tendency to be present more often in women with a known history of cardiovascular disease than in women without a history of cardiovascular disease (70%, *n* = 9, vs 40%, *n* = 17, *p* = 0.06, odds ratio (OR) 3.44, 95% confidence interval (CI) 0.91–12.97).

**Table 1:** Baseline characteristics, time and causes of death for the women who died during pregnancy or in the postpartum period due to cardiovascular diseases in 1993–2013 in the Netherlands

Period	Total (1993–2013)	1st decade (1993–2003)	2nd decade (2003–2013)^a^
*Number of women*	96	36	60
*Age* (mean years, SD)	31.6 (5.8)	30.4 (6.1)	32.4 (5.6)
*Gravidity* (median, range)	2 (1–11)	2 (1–11)	2 (1–7)
*Parity* (median, range)	1 (0–5)	1 (0–5)	1 (0–4)
*Time of death*			
During pregnancy: 1st trimester 2nd trimester 3rd trimester	7 (7%) 11 (11%) 16 (16%)	2 (6%) 4 (11%) 8 (22%)	5 (8%) 7 (12%) 8 (13%)
During postpartum period: <21 days >21 days	22 (22%)33 (33%)	8 (22%)11 (31%)	14 (23%)23 (38%)
Unknown	7 (7%)	3 (8%)	3 (5%)
*Ethnicity*			
Dutch	60 (63%)	21 (58%)	39 (65%)
Surinamese/Dutch Antilles	9 (9%)	6 (17%)	3 (5%)
Turkish	5 (5%)	3 (8%)	2 (3%)
Other	6 (6%)	3 (8%)	3 (5%)
Unknown	16 (17%)	3 (8%)	13 (22%)
*Cause of death*			
SUDS/SADS	27 (28%)	7 (19%)	20 (33%)
Aortic dissection	20 (21%)	7 (19%)	13 (22%)
Ischaemic heart disease	17 (18%)	9 (25%)	8 (13%)
*– of which acute myocardial infarction*	*14 (15%)*	* 7 (19%)*	* 7 (12%)*
Cardiomyopathy, not pregnancy-related Pregnancy-related Myocarditis Unknown	8 (8%) 4 (4%) 7 (7%) 1 (1%)	3 (8%) 2 (3%) 3 (8%)–	6 (10%) 2 (6%) 4 (7%) 1 (2%)
*Other*	12	5	7
PHV complications	4 (4%)	2 (6%)	2 (3%)
Valvular heart disease (no PHV)	4 (4%)	2 (6%)	2 (3%)
Congenital heart disease	1 (1%)	1 (3%)	–
Pulmonary hypertension	1 (1%)	–	1 (2%)

*VF* ventricular fibrillation, *PHV* prosthetic heart valve, *SADS* sudden adult death syndrome, *SUDS* sudden unexplained death syndrome

^a^Includes the year 2003

**Table 2:** Possibly avoidable care factors contributing to maternal death in women who died due to cardiovascular disease during 1993–2013 in the Netherlands

Period	Total (1993–2013)	1st decade (1993–2003)	2nd decade (2003–2013)
**Total reported cases of substandard care (*n*, *%*)**	*27 (28%)*	*12 (33%)*	*15 (25%)*
*Healthcare-provider-related*			
Correct diagnosis not made by doctor or midwife	12 (44%)	6 (50%)	6 (40%)
Delay in referral to specialist	4 (15%)	2 (17%)	2 (13%)
*Patient-related*			
Pregnancy initiated against medical advice	6 (22%)	2 (17%)	4 (27%)
No acknowledgement of complaints by patient	3 (11%)	1 (8%)	2 (13%)
Discontinuation of medication against medical advice	2 (7%)	1 (8%)	1 (7%)

Thirty-four women died during pregnancy. Their offspring all died (*n* = 33, including two twin pregnancies). Fifty-five women died postpartum (58%). There were three perinatal deaths in three of these women and one early foetal death after successful maternal cardiopulmonary resuscitation. Median timing of delivery was 37 + 2 weeks (range 22–42 weeks); mean birth weight was 2709 g (SD 914 g).

Reported obstetric and perinatal complications are presented in Tab. 3. There were no cases of eclampsia or HELLP syndrome. CS (*n* = 10) was performed for maternal indications in five planned and five emergency cases.

**Table 3:** Obstetric and perinatal complications in pregnancies in women who died due to cardiovascular disease in the Netherlands

Period	Total (1993–2013)	1st decade (1993–2003)	2nd decade (2003–2013)
**Total reported pregnancies with obstetric complications (*n*, *%*)**	*33 (34%)*	* 9 (25%)*	*24 (39%)*
Planned CS	7 (7%)	1 (6%)	6 (18%)
Emergency CS	10 (19%)	4 (24%)	6 (18%)
Abortion	1 (2%)	1 (5%)	0
PIH	4 (5%)	2 (8%)	2 (4%)
Pre-eclampsia	6 (8%)	2 (8%)	4 (8%)
PPH	7 (13%)	2 (11%)	5 (15%)
Hyperemesis (pregnancy-induced)	2 (3%)	–	2 (4%)
Gestational DM	1 (1%)	0	1 (2%)
Preterm labour	3 (6%)	1 (6%)	2 (6%)
**Total reported pregnancies with offspring complications (*n*, %)**	*55 (55%)*	*22 (60%)*	*33 (52%)*
Early foetal death	15 (17%)	5 (16%)	10 (19%)
*– accompanied by maternal death (% of total)*	*14 (93%)*	* 5 (100%)*	* 9 (90%)*
Perinatal death	22 (27%)	10 (35%)	12 (23%)
*– accompanied by maternal death (% of total)*	*19 (86%)*	* 9 (90%)*	*10 (83%)*
Prematurity	17 (35%)	7 (47%)	10 (29%)
Low birth weight	12 (29%)	4 (29%)	8 (29%)
NRDS	1 (2%)	1 (7%)	–
Apgar <7	1 (3%)	–	1 (4%)

*CS* Caesarean section, *DM* diabetes mellitus, *NRDS* neonatal respiratory distress syndrome, *PIH* pregnancy-induced hypertension, *PPH* postpartum haemorrhage

Missing data are excluded from analysis except for total reported numbers of complications

### SUDS and SADS

Twenty-seven women died due to unexplained or arrhythmic sudden death, SUDS (*n* = 19) or SADS (*n* = 8).

Of the women who died due to SADS, two were known to have cardiac rhythm disorders pre-pregnancy. In both women contributing care factors were identified. One woman with a long QT syndrome had discontinued her beta-blocker therapy against medical advice. The other woman was known to have Wolf-Parkinson-White syndrome. She had been arrhythmia-free without medication for 6 years. There was a delay in referral to a cardiologist when she complained about palpitations during pregnancy. The other six women who died due to SADS were either not diagnosed or not known to have been diagnosed with arrhythmias pre-pregnancy.

Autopsy was definitely not performed in 12 of 19 women with SUDS and not performed or not reported to the MMMC in another four cases. Five women had cardiac or non-cardiac underlying diseases that may possibly but not definitely have been related to the sudden death (myocardial infarction and heart failure, aortic valve surgery, left ventricular hypertrophy and anomalous left coronary artery, hyperthyroid disease, hyperemesis with dehydration).

Time of death was during pregnancy in eight and during the postpartum period in 16 women. Maternal death accompanied intrauterine and early foetal death in seven cases. Delay in hospital referral was reported in one woman. Information about possible contributing suboptimal care factors was missing in 22 cases.

### Aortic dissection

Twenty women (21%) died from aortic dissection. Most dissections occurred in the third trimester (40%) and postpartum (35%). Pre-existing relevant morbidity was present in eight women (40%): three had a connective tissue disease (18% compared to 0% in women who died due to other CVD, *p* < 0.01) and five had pre-pregnancy hypertension (25%). Hypertensive obstetric complications occurred in four women (pregnancy-induced hypertension (*n* = 3) and pre-eclampsia (*n* = 1)). Overall, seven women had hypertension, pre-existent or pregnancy-induced (41% in women with aortic dissection vs 19% in women who died due to other CVD, *p* = 0.052). Maternal death accompanied perinatal death in 11 cases. In eight women (40%) suboptimal care factors were identified. In seven cases these involved the presence of signs and symptoms of aortic dissection (including chest or back pain, dyspnoea and circulatory failure) without this leading to proper diagnostic tests and correct diagnosis. Two of these women were misdiagnosed with a psychiatric disorder. In one case the diagnosis was rejected after false-negative sonography.

### Ischaemic heart disease

Seventeen women (18%) died from ischaemic heart disease (IHD). None of these women were known to have coronary artery disease. In three women the diagnosis of IHD was made at autopsy; two of them presented with heart failure. Fourteen women (13% of the total number of maternal deaths) presented with acute myocardial infarction (AMI) during pregnancy. All but one woman presented during the third trimester or postpartum period (1 case of missing data). AMI was caused by coronary dissection (*n* = 4), coronary sclerosis/thrombosis (*n* = 2) or other/unspecified causes. Risk factors for IHD were reported in four women (33%). Obstetric events included postpartum haemorrhage (*n* = 3, not related to maternal death) and CS because of pre-eclampsia (*n* = 2). Maternal death accompanied neonatal death (*n* = 5) in four cases. In five cases underestimation of the complaints was reported (four times by the doctor, once by the patient).

### Cardiomyopathy

Thirteen women (14%) died from cardiomyopathy. In 43% potentially avoidable contributing care factors were identified.

#### Peripartum cardiomyopathy

The four women with peripartum cardiomyopathy all died postpartum. One of these pregnancies had been complicated by pre-eclampsia and delivery of a child with a low birth weight at 37 weeks. There were no other obstetric or neonatal complications. In one woman there was a delay in diagnosis; another woman discontinued her medication against medical advice.

#### Other types of cardiomyopathy

Ten women died from cardiomyopathies other than peripartum cardiomyopathy. Three had dilated cardiomyopathy, three arrhythmogenic cardiomyopathy, one hypertrophic cardiomyopathy and one Takotsubo cardiomyopathy after a difficult vaginal delivery. Most women died in the postpartum period (*n* = 8, data missing in 1 case). Three of them had been diagnosed before pregnancy. Four of the seven women who were not known to have cardiomyopathy had a positive first-degree family history of cardiomyopathy (*n* = 2) or acute death in a young first-degree relative (<45 years, *n* = 2). Whether or not pre-pregnancy screening had been performed in these women is unknown. Obstetric complications were reported in three pregnancies and included one planned and one emergency CS. Neonatal complications were observed in four pregnancies, including one early foetal death. Contributing substandard care factors were reported in four cases (44%, data missing in 1 case): pregnancy against medical advice (*n* = 2), delay in referral to a specialist (*n* = 1) and underestimation of the complaints by the patient (*n* = 1).

Seven women (7%) died from myocarditis, five during pregnancy and one postpartum (1 time of death missing). In five women the diagnosis was made at autopsy (unknown in 2 cases). Myocarditis was caused by auto-immune disorders (*n* = 3) or unknown causes (*n* = 2). In two cases the distinction from peripartum cardiomyopathy was not fully clear. None of the women had a history of cardiac disease. There were no obstetric complications. Maternal death was accompanied by offspring death in three cases. In one woman a delay in referral to a specialist was reported.

### Other CVD

#### Valvular heart disease and prosthetic heart valves

Four women died due to native valvular heart disease; three women became pregnant against medical advice. One woman, known to have severe aortic and mitral regurgitation, had refused cardiac surgery and died suddenly during the postpartum period. A woman with congenital aortic stenosis and mitral regurgitation died due to decompensated aortic stenosis in the third trimester; her foetus also died. Two women died due to complicated endocarditis.

Four women (4%) died due to complications of their prosthetic heart valves (PHVs). Three deaths were attributable to mechanical PHV thrombosis. All women were treated with low-molecular-weight heparin (LMWH) at the time of valve thrombosis. One woman died 2.5 months after cardiogenic shock and resuscitation due to a thrombosed aortic PHV in the second trimester of pregnancy. Her foetus died as well. Another woman had a thrombosis of her mitral PHV in the third trimester of pregnancy. Acute re-operation was complicated by cardiac tamponade, sepsis, intracranial haemorrhage and brain-stem herniation. The third woman died in the first trimester due to heart failure; a thrombosed mitral PHV was observed on autopsy. Substandard care was not reported to the MMMC. Whether or not anti-Xa measurements were performed during LMWH treatment was not reported to the MMMC. These cases of PHV thrombosis occurred in 2002, 2008 and 2008.

One other PHV-related death occurred due to haemorrhagic complications of anticoagulation therapy during the third trimester. The woman died due to pulmonary haemorrhage while she was being anticoagulated with a vitamin‑K antagonist (VKA) (INR 7.5). This woman was pregnant against medical advice. Her child died as well.

### CHD and pulmonary hypertension

One woman died from CHD and one from pulmonary hypertension. A woman with a Fontan circulation died due to cardiovascular collapse postpartum after a CS for a maternal non-cardiac indication. She had been advised against pregnancy.

A 28-year-old primiparous woman with systemic lupus erythematosus died 3 months postpartum due to heart failure caused by previously unrecognised pulmonary hypertension.

## Discussion

Our study showed that in the last 21 years the majority of maternal deaths due to cardiovascular disorders in the Netherlands were caused by aortic dissection, IHD, cardiomyopathies and SUDS/SADS. Interestingly, these cardiovascular causes of maternal death are very similar to those reported in the UK in the last 3 years (Fig. 1; [16]). Aortic dissection, IHD and cardiomyopathy are rare diseases in young pregnant women, often presenting acutely and unexpectedly. Additionally, the presentation in pregnant women can be atypical or symptoms may be attributed to normal pregnancy discomforts. This likely contributes to the high mortality of these diseases in pregnant women. Many deaths occurred postpartum. Physicians and first-care providers should realise that after the early postpartum period women remain at increased risk of fatal pregnancy-related complications.

Unfortunately, the incidence of possibly avoidable care factors that may have contributed to the adverse outcomes did not significantly decrease when comparing the period 1993–2003 with 2003–2013. This may be partly attributable to the slight rise in patient-related factors, which underlines the importance of adequate pre-pregnancy counselling of women with known heart disease. A major contributing factor related to healthcare provision was failure to make the diagnosis in time. This calls for extended education of caregivers concerning recognition and treatment of cardiac diseases during pregnancy and the postpartum period [2, 3].

Also, maternal death occurred more frequently in women with a Dutch-Antillean or Surinamese background. This may possibly be explained by the higher incidence of risk factors for CVD in these women, such as hypertension [17].

### SUDS and SADS

As in other recent reports, the largest group of contemporary maternal cardiac deaths were unexplained sudden deaths (SUDS and SADS) [1]. Whether or not these deaths are definitely from cardiac causes is therefore not sure. In our study autopsy was frequently not performed in those women, which is unfavourable with regard to medical knowledge and science and may in some cases even be legally disputable in the Netherlands. Failure to perform autopsy was not considered by the MMMC to represent substandard care during the period studied, since it did not influence maternal death. However, it may contribute to suboptimal care for the families of these deceased mothers because positive findings at autopsy facilitate family screening and may eventually contribute to preventing further deaths in these families. Four women with SUDS had conditions which may have induced cardiac arrhythmias. Women with known cardiac rhythm disorders should be under cardiological supervision before and during pregnancy. In some women, a switch in anti-arrhythmic medication to avoid foetal risk associated with some anti-arrhythmic medication may be necessary [18]. When a woman with a cardiac rhythm disorder experiences palpitations, dizziness, dyspnoea or chest pain during pregnancy, she should be referred to a cardiologist without delay for further evaluation.

### Aortic dissection

Aortic dissection during pregnancy is rare but has a high case fatality rate of 83% [19]. The diagnosis can be challenging and it should be considered as a possible cause of thoracic pain, especially when risk factors for aortic dissection (e.g. connective tissue disease or hypertension) are present, as in 50% of our population [20]. Despite the presence of symptoms indicative of aortic dissection the diagnosis has frequently not been made [21]. Interestingly, two women were misdiagnosed with a neuropsychiatric disorder, which has been described as a pitfall in the presentation of aortic dissection [22–24]. Sonography alone is insufficient to exclude aortic dissection according to current guidelines, as is illustrated by one of our cases [21]. We recommend that aortic dissection should be considered in every pregnant woman with risk factors for aortic dissection and unexplained chest pain, back pain, also when combined with unexplained neuropsychiatric symptoms. Electrocardiography (ECG)-gated computed tomography of the chest is indicated when there is a reasonable suspicion of aortic dissection.

### Ischaemic heart disease

IHD was the second most frequent cause of maternal cardiac death, in line with other reports [1]. Pregnancy is known to increase the risk of manifestations of IHD 3‑ to 4‑fold [25]. Acute coronary syndromes are known to be more prevalent during late pregnancy [26]. The maternal mortality ratio is 3–11% and maternal morbidity, foetal mortality and morbidity rates are also high [19, 26, 27]. Consistent with other reports we found coronary dissection to be a frequent cause of IHD [26]. Risk factors for coronary artery disease are often present, especially in women with atherosclerotic disease [26]. Proper recognition of the diagnosis appeared difficult and the severity of complaints was often underestimated. In pregnant women with chest pain it is not adequate only to rule out the differential diagnosis of pulmonary embolism and aortic dissection. IHD should be ruled out by ECG and biomarker tests. It is important to realise that IHD can also have an atypical presentation with symptoms such as syncope or dyspnoea.

### Cardiomyopathy

A recent national study found a high incidence of cardiovascular morbidity due to cardiomyopathy during pregnancy but with a relatively low case fatality rate [19]. In our study cardiomyopathy was the third most frequent cause of maternal cardiac death. Four women died due to peripartum cardiomyopathy, which is known for its high mortality risk up to 30% [28]. Care factors related to the adverse outcome were mostly patient-related. Interestingly, one woman died from Takotsubo cardiomyopathy, which is a rare diagnosis during pregnancy. It is a stress-related cardiomyopathy presenting with severe heart failure and chest pain, with typical wall motion abnormalities of the apical region (apical ballooning). During pregnancy it has been mainly related to CS or pre-eclampsia. However, in our patient the Takotsubo cardiomyopathy occurred after a difficult vaginal delivery [29]. One woman died due to hypertrophic cardiomyopathy, a condition usually well tolerated during pregnancy with low mortality rates (0.5%) [30]. The number of patients with myocarditis is remarkable. In two cases the differential diagnosis of peripartum cardiomyopathy was not definitely ruled out.

Half of the women who were not known to have (non-pregnancy-related) cardiomyopathy before pregnancy had a positive family history for cardiomyopathy or early sudden death. These are the women who may benefit from pre-pregnancy screening and cardiac evaluation including echocardiography. Physicians should always consider family screening in first-degree relatives of patients with cardiomyopathy or early sudden death. Furthermore, in newly pregnant women a family history should be taken and women with a positive family history for cardiomyopathy and/or acute death should be evaluated by a cardiologist early during the pregnancy if screening has not been performed pre-pregnancy. Women with known cardiomyopathy should be under tight medical supervision during pregnancy as well as during the postpartum period.

### Other CVD

Valvular heart disease is a frequent cause of cardiovascular morbidity during pregnancy in the Netherlands but has relatively low case fatality rates [19]. However, the cases described illustrate that the risk of severe native valvular disease should not be underestimated. Most women with valvular disease who died had been advised against pregnancy. Women who are advised against pregnancy for medical reasons may need intensive psychological and medical support and should be advised about safe and effective contraception. In women with a mechanical PHV, the main concern during pregnancy is balancing the risk of PHV thrombosis (which is relatively high with LMWH therapy) with the risk of bleeding and the risk of embryopathy (which exists only with warfarin therapy during the first trimester but not with LMWH therapy). Thrombosis and bleeding were causes of death in our population. All women who died due to PHV thrombosis were anticoagulated with LMWH. A high maternal death rate (9%) in women with a mechanical PHV has recently been described in the UK and seemed to be related to LMWH [31]. Given the higher risk of valve thrombosis, current European and American guidelines advise that LMWH therapy should be limited to the first trimester and the last month of pregnancy, and should be used only when frequent anti-Xa monitoring with dose-adjusting is available. In women needing a low dose of a VKA, continuing with the antagonist throughout pregnancy should be considered [32, 33]. Data concerning anti-Xa monitoring were not available to the MMMC and not taken into account in the assignment of substandard care. It is important to realise that the anticoagulation dose can change during pregnancy and therefore monitoring of the anticoagulation effect is more frequently necessary than pre-pregnancy or during the postpartum period [32, 33].

Though CHD is the most frequent underlying maternal heart disease in the Western world, only one death occurred, in a woman advised against pregnancy. The woman who died due to unrecognised pulmonary hypertension was known to have a condition associated with pulmonary hypertension (systemic lupus erythematosus). Pre-pregnancy evaluation in women with these conditions may influence pre-pregnancy advice, while current guidelines still advise against pregnancy in women with pulmonary hypertension because of high maternal mortality and morbidity rates [33].

## Limitations

Data and analysis were limited by the amount of available data delivered to the MMMC. While we choose to cross-check cases of maternal death with vital data from Statistics Netherlands to prevent missing such a case, we deprived ourselves of additional data which could not be provided or retrieved by Statistics Netherlands. Missing data could therefore not be prevented. Finally, while expert help was obtained when needed, a cardiologist and an acute care specialist such as an emergency physician were not systematically involved in the evaluation of cases of maternal death by the MMMC.

## Conclusion and key recommendations

Maternal cardiovascular mortality rates are low in the Netherlands. In a minority of cases, possibly avoidable care factors (attributable to the patient or the healthcare provided) contributed to the adverse outcome. Our data result in several recommendations that may contribute to further improvement of care for pregnant women with cardiovascular diseases.

- Women with known heart disease should be advised about pursuing pregnancy, following current guidelines, and should be closely monitored by a cardiologist and a perinatologist with expertise in the field of pregnancy and heart disease.
- Awareness should be raised about the postpartum period being a vulnerable period for maternal cardiac death. Not all chest pain in pregnant women is caused by pulmonary embolism. Chest pain or back pain, especially when hypertension or a family history of connective tissue disorders is present, may indicate aortic dissection and computed tomography of the chest should be considered.
- Ischaemic heart disease should be considered in pregnant women with chest pain, and ECG and a cardiac biomarker test should be performed.
- Women with a positive family history for cardiomyopathy and/or acute death should be evaluated pre-conceptionally by a cardiologist.
- Women with known cardiomyopathy should be under tight medical supervision during pregnancy as well as the whole postpartum period.
- Women with a heart valve prosthesis should be treated during pregnancy in an expert centre according to guidelines under close anti-Xa or INR (self) monitoring depending on the type of anticoagulation, in order to prevent PHV thrombosis or bleeding complications [32, 34].
- Autopsy should always be performed in women who die from unexplained causes during pregnancy or in the postpartum period.
